# Hospital‐acquired infections as a risk factor for post‐traumatic epilepsy: A registry‐based cohort study

**DOI:** 10.1002/epi4.12957

**Published:** 2024-05-10

**Authors:** Zhibin Chen, Joshua Laing, Jian Li, Terence J. O'Brien, Belinda J. Gabbe, Bridgette D. Semple

**Affiliations:** ^1^ Department of Neuroscience, School of Translational Medicine Monash University Melbourne Victoria Australia; ^2^ Department of Medicine, The Royal Melbourne Hospital The University of Melbourne Melbourne Victoria Australia; ^3^ Department of Neurology, The Royal Melbourne Hospital The University of Melbourne Melbourne Victoria Australia; ^4^ School of Public Health and Preventive Medicine Monash University Melbourne Victoria Australia; ^5^ Epilepsy Unit Alfred Hospital Melbourne Victoria Australia; ^6^ Department of Neurology Peninsula Health Melbourne Victoria Australia; ^7^ Biomedicine Discovery Institute and Department of Microbiology Monash University Melbourne Victoria Australia; ^8^ Alfred Health Prahran Victoria Australia; ^9^ Health Data Research UK Swansea University Swansea UK

**Keywords:** bacterial, epilepsy, meningitis, nosocomial, seizure, sepsis

## Abstract

**Objective:**

Hospital‐acquired infections are a common complication for patients with moderate or severe traumatic brain injury (TBI), contributing to morbidity and mortality. As infection‐mediated immune responses can predispose towards epilepsy, we hypothesized that post‐injury hospital‐acquired infections increase the risk of post‐traumatic epilepsy (PTE).

**Methods:**

A retrospective cohort study of adults with moderate to severe TBI was conducted using data from the Victorian State Trauma Registry in Australia. Infections were identified from the International Statistical Classification of Diseases and Related Health Problems 10th Revision–Australian Modification (ICD‐10‐AM) codes, and diagnosis of PTE was determined by the Glasgow Outcome Scale – Extended questionnaire regarding epileptic fits at 24 months follow‐up.

**Results:**

Of all TBI patients (*n* = 15 152), 24% had evidence of having had any type of infection, with the most common being pneumonia, urinary tract, and respiratory infections. Of those who responded to the PTE question at 24 months (*n* = 1361), 11% had developed PTE. Univariable analysis found that the incidence of PTE was higher in patients who had any type of infection compared to patients without an infection (*p* < 0.001). After adjustment for covariates associated with both development of PTE and risk of infection, multivariable analysis found a solid association between infection and PTE (adjusted RR = 1.59; 95% CI: 1.11–2.28; *p* = 0.011). Having any type of complicating infection acquired during admission was also associated with poor GOSE outcomes at subsequent follow‐ups (adjusted OR = 0.20; 95% CI: 0.11–0.35, *p* < 0.001).

**Significance:**

These findings suggest that hospital‐acquired infections contribute to PTE development after TBI. Future investigation into infections as a modifiable target to reduce poor outcomes after TBI is warranted.

**Plain Language Summary:**

Hospital‐acquired infections are common in patients with traumatic brain injuries. A database study of adults with moderate or severe brain injuries in Australia examined whether these infections are associated with the development of epilepsy after a brain injury. 24% of patients had infections, with pneumonia and urinary tract infections being the most common. Of those surveyed 2 years after the injury, 11% developed post‐traumatic epilepsy. Patients with infections had a significantly higher risk of epilepsy, even when accounting for other known risk factors, and infections were also linked to poor outcomes more broadly. The study suggests that preventing hospital‐acquired infections could be a crucial target for improving outcomes after traumatic brain injuries.

**Key points**

- Post‐traumatic epilepsy (PTE) is a common consequence of traumatic brain injuries.
- We explored whether the risk of PTE was altered by a prior hospital‐acquired infection.
- Univariable analysis found a higher incidence of PTE in those who had sustained an infection.
- The association remained after adjustment for covariates such as injury severity.
- Future studies to explore this potential relationship are warranted.

## 1 INTRODUCTION

Post‐traumatic epilepsy (PTE), defined as unprovoked seizures at least seven days after the initial injury,^1^ is a common consequence of traumatic brain injury (TBI).^2^ For people who develop PTE, higher mortality and morbidity have been reported, including an increased likelihood of neurological and psychological co‐morbidities, resulting in a significant additional health burden.^3^, ^4^, ^5^ Prevention or suppression of PTE requires an understanding of the risk factors that contribute to its development, a process known as epileptogenesis. Risk factors identified to date include injury severity (low presenting Glasgow Coma Scale [GCS] score),^6^, ^7^, ^8^ presence of a skull fracture,^7^ penetrating or additional craniofacial injuries,^9^ acute post‐traumatic seizures,^9^, ^10^ and genetic variation.^11^, ^12^ Another potential risk factor unexplored in this context is post‐injury infection.

Infections are common complications for patients with TBI during hospitalization.^13^ Up to 50% of patients with severe TBI admitted to intensive care units are reportedly affected by nosocomial infections.^14^, ^15^, ^16^ The high incidence is multifactorial, primarily attributable to the need for mechanical ventilation, urinary catheters, and other invasive medical/surgical procedures that may introduce an infection source.^13^ Furthermore, reported immunosuppression involving acute lymophopenia and a dysregulated T cell‐mediated response after TBI may contribute to increased vulnerability to infections.^14^, ^15^ Hospital‐acquired pneumonia and sepsis, in particular, are established contributors to poor outcomes after TBI, leading to increased mortality, longer hospitalizations, increased ventilator use, a higher risk of organ failure, and a poorer functional outcome compared to TBI patients who do not sustain an infection.^17^, ^18^, ^19^, ^20^, ^21^

This evidence raises the question of whether infections might also promote the development of PTE. Mechanistically, inflammatory immune cells and mediators such as pro‐inflammatory cytokines may be upregulated in response to an infection, and have been proposed to play a central role in seizure provocation as well as epileptogenesis by directly and indirectly promoting neuronal hyperexcitability.^22^, ^23^, ^24^ Independent of TBI, infections are well‐known contributors to seizures and epilepsy, such as febrile seizures during infancy.^23^, ^25^ In adults, a large study of almost 2 million Danish individuals found that hospital contact with infection (primary diagnosis of infection) was associated with a 78% increase in the risk of a subsequent epilepsy diagnosis compared to people without exposure to infection.^22^

After TBI, we hypothesize that the additional insult of sustaining an infection may drive the development of PTE in an injured brain that is already at a higher risk of epilepsy as a result of the injury. However, very few studies have considered a potential link between infectious complications and PTE, and most relate to the specific context of penetrating head injuries in military settings.^26^, ^27^ Limited preclinical studies incorporating inoculation of live bacteria with experimental TBI models typically support the hypothesis that infections worsen outcomes [as recently reviewed in Ref. 28], although seizures and epilepsy have not been explored in this context to date. Clinically, a clear association between infections (while hospitalized) and PTE in TBI patients has not been reported, nor has this potential association been examined in a large civilian population of individuals with moderate to severe TBI. Here, we therefore used an Australian population‐based trauma registry to test the hypothesis that sustaining an infection during the acute hospitalization period following a moderate to severe TBI would increase the risk of PTE at 2‐year follow‐up.

## 2 METHODS

### 2.1 Sample population

This was a registry‐based cohort study of adult patients (≥18 years of age) admitted with a moderate to severe TBI to trauma‐receiving hospitals in the state of Victoria, Australia, with a date of injury between January 2005 to December 2019, as recently reported.^10^, ^29^ Patient data was sourced from the Victorian State Trauma Registry (VSTR) with follow‐up till 10 May 2020. The registry has been collecting data Victoria‐wide since July 2001 and has had complete population capture since July 2005.

**FIGURE 1:**
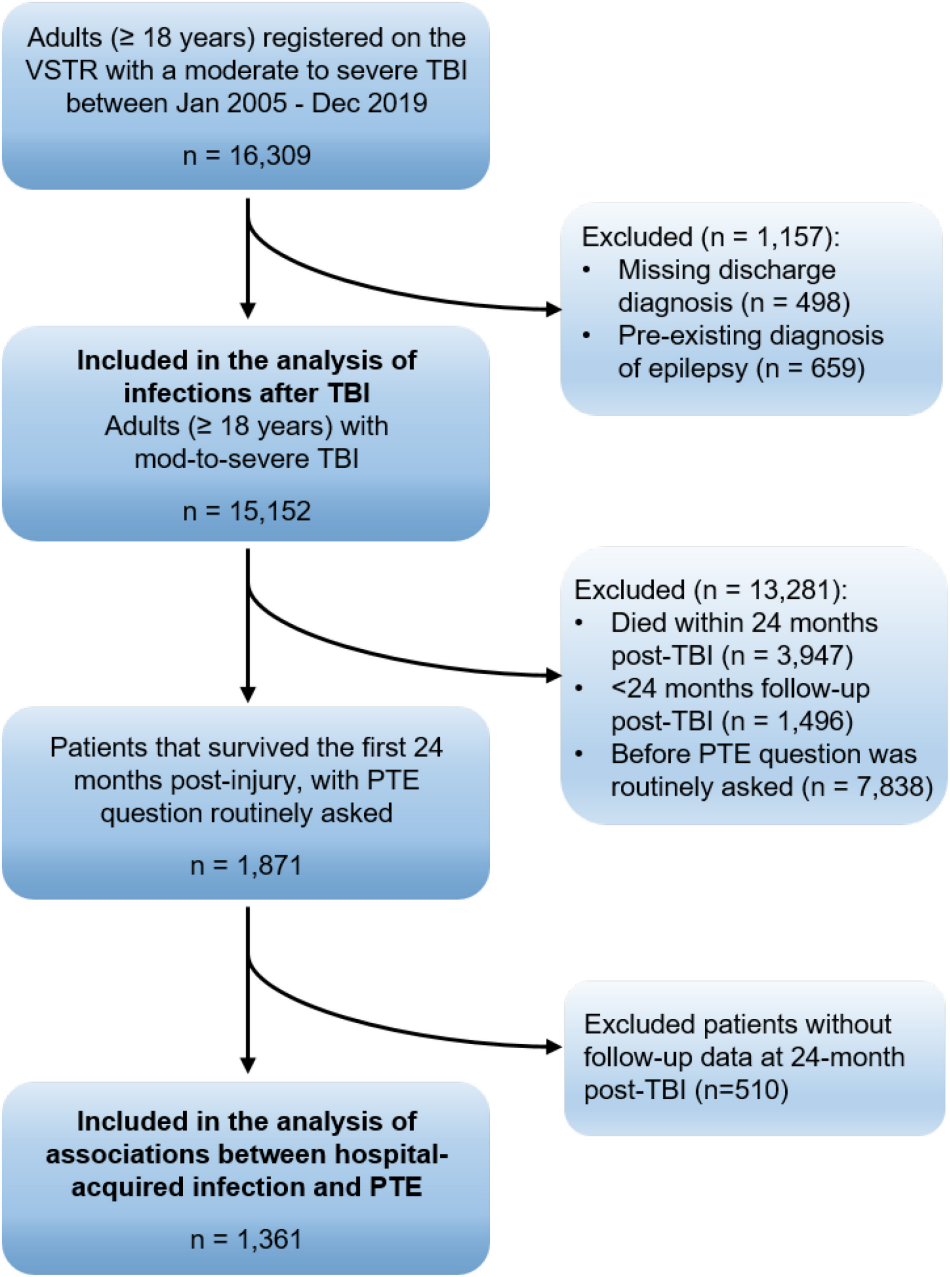
STROBE flow chart, illustrating included and excluded patients with moderate to severe traumatic brain injury (TBI) from the Victorian State Trauma Registry (VSTR), to perform an analysis on potential associations between hospital‐acquired infections and post‐traumatic epilepsy (PTE). STROBE = Strengthening the Reporting of Observational Studies in Epidemiology, as per von Elm et al.^33^

Moderate to severe TBI was defined as an Abbreviated Injury Scale (AIS) head severity score of 3 (serious, non‐life threatening) to 6 (unsurvivable), indicating moderate to severe intracranial injury or skull fracture.^30^ The AIS is an anatomical injury severity scoring system with established correlation with TBI outcomes,^30^ which reportedly correlates poorly with the clinical examination‐based GCS.^31^ However, some studies have reported that head AIS injury categories are better able to cluster TBI patients into homogenous groups, reflecting its superior ability to indicate injury severity.^32^ We also focused on the AIS head score as it reflects a confirmed intracranial injury or skull fracture, while GCS is a measure of consciousness which can be impaired even in the absence of intracranial injury. Patients with a pre‐existing diagnosis of epilepsy were excluded from the study, as identified using the International Statistical Classification of Diseases and Related Health Problems 10th Revision–Australian Modification (ICD‐10‐AM) codes from the admission. Reporting of patient inclusion/exclusion is in alignment with the Strengthening the Reporting of Observational Studies in Epidemiology (STROBE) guidelines (Figure 1).^33^

### 2.2 Standard protocol approvals, registrations, and patient consents

The VSTR dataset was collected with ethical approval from the Victorian Department of Health and Human Services Human Research Ethics Committee (Project ID #78938) and the Monash University Human Research Ethics Committee (CF13/3040‐2001000165). An anonymized dataset was extracted from the VSTR of eligible patients following project‐specific ethics approval from Monash University's Human Ethics Committee (MUHREC Project ID 18104).

### 2.3 Data collection

ICD‐10‐AM codes were completed at discharge for the period of admission. Extracted data included demographics (e.g., age, sex), injury‐related information (e.g., polytrauma, cause of injury, type of injury), and discharge information including in‐hospital mortality and comorbidities (e.g., history of drug or alcohol abuse). In addition, ICD‐10‐AM diagnosis related to infections of various specified and unspecified causes were extracted (e.g., sepsis, meningitis, pneumonia, wound infection) where coded as complicating diagnoses (prefix C), indicating that the condition(s) were acquired following admission/primary diagnosis. Included ICD‐10‐AM codes are presented in Table 1. As previous literature has suggested that pneumonia is the most common complicating infection in this high‐risk patient group,^13^, ^34^, ^35^ we considered this as a distinct category as well as grouping all “respiratory infections” together for analysis.

**TABLE 1:** ICD‐10‐AM codes for the corresponding conditions included in the analysis.

Conditions	ICD‐10‐AM
Intestinal infections	A00.X–A09.X
Sepsis	A40.X; A41; A41.5; A41.9; R57.2; T81.42
Non‐specified bacterial infections	A49.X
Meningitis	A87.X; G03; G03.0; G03.1; G03.8; G03.9
All respiratory infections	J00.X–J06.X; J20.X–J22.X; J69.0; J69.8
Upper respiratory	J00.X–J06.X
Lower respiratory	J22.X; J69.0; J69.8
All pneumonia	J12.X; J15.X; J16.X; J18.X
Urinary tract infections	N39.0; T83.5
Fever	R50.9
Post‐traumatic infections	T79.3
Wound infections	T80.2; T81.4; T81.41

*Note*: The. X suffix indicates all the sub‐classifications (where applicable) of the category are included.

Isolated TBI was defined as the absence of an injury in any other AIS body region with a severity score > 1. The Injury Severity Score (ISS) is derived from the AIS to provide an overall severity score.^30^ The initial Glasgow Coma Scale (GCS) score was used as a further measure of TBI severity. The Charlson Comorbidity Index (CCI) and indicators for pre‐existing mental health, drug, and alcohol conditions were also mapped from the ICD‐10‐AM codes.^36^, ^37^ The Accessibility and Remoteness Index of Australia (ARIA), a measure of geographical remoteness, was determined by residential postcode and was used as an indicator of accessibility to services including healthcare. Early seizures were defined based on the ICD‐10‐AM codes R56.8 (unspecified convulsions; 91.6%), G41 (status epilepticus) and G40 (acute symptomatic seizures), to identify new onset seizures during the acute admission stage but exclude pre‐existing epilepsy, as previously reported.^10^

TBI cases on the VSTR were routinely followed up by standardized, structured telephone interviews at 6, 12, and 24 months post‐injury, for collection of the Glasgow Outcome Scale‐Extended (GOS‐E).^38^ For the purposes of this study, data from all three follow‐up time points were accumulated, and PTE status was analyzed at the 24‐month time point. This decision was based upon previous studies indicating a higher incidence of PTE as more time passes after a TBI, up to at least 2 years.^39^, ^40^ Diagnosis of PTE was determined by patient responses to the GOS‐E question: “Since the injury, has the patient had any epileptic fits?”.^38^ The question was routinely asked at all follow‐up interviews from 12 March 2018. We thus considered a PTE diagnosis according to the most recent ILAE definition, where epilepsy can be diagnosed after *“One unprovoked (or reflex) seizure and a probability of further seizures similar to the general recurrence risk (at least 60%) after two unprovoked seizures, occurring over the next 10 years.”* ^1^ Previous studies have found that up to 86% of patients who have had a first late seizure following a moderate–severe TBI will have recurrent seizures within two years.^2^, ^41^

### 2.4 Statistical analysis

Descriptive statistics for hospital‐acquired infections included all patients who met the inclusion criteria. Frequencies and percentages for categorical variables, and median and interquartile ranges (IQR) for non‐normally distributed continuous variables, were used to summarize the data. Analysis of the incidence of PTE at 24 months follow‐up was limited in patients who were due to have 24 months follow‐up on 12 March 2018 or afterward. Univariable associations between any complicating infection or infection subtypes and development of PTE were assessed using chi‐square test. Any complicating infection and all the infection subtypes with *p*‐value < 0.25 in the univariable analysis were then put into multivariable Poisson regression models with robust variance, separately.^42^ From a list of demographic and clinical factors that were described in our previous study,^10^ we used purposeful variable selection method^42^ to select potential confounders that might be associated with both development of PTE (the outcome of interest) and the risk of any complicating infection acquired during admission (the exposure of interest). Factors that had univariable *p*‐value < 0.25 with both development of PTE and the risk of any complicating infection were selected as covariates and included in the initial model. The Bayesian Information Criterion (BIC) and Akaike Information Criterion (AIC) were calculated for all possible combinations of covariates, where lower BIC and AIC values indicate preferable combinations. To minimize overfitting while maintaining a relatively good model fit, a two‐step model selection approach was employed. Firstly, combinations with the smallest BIC values and which were not significantly different (defined as having an absolute difference of less than 2) were shortlisted. Subsequently, the combination with the lowest AIC was chosen for inclusion in the multivariable analyses. Adjusted risk ratios (RR) and the corresponding 95% confidence intervals (CIs) were reported. Mixed‐effect multilevel ordinal logistic regression with random intercept at patient level was used to assess the effect of any complicating infection on recovery outcomes GOSE, with adjustments of relevant covariates including follow‐up time point, early posttraumatic seizures, age, sex, history of mental health conditions, and Glasgow Coma Scale category. The statistical significance level was set at *p* < 0.05. All statistical analyses were performed using *Stata* version 16.1 (StataCorp).

## 3 RESULTS

### 3.1 Frequency of hospital‐acquired infections after TBI

Over the 15‐year period of captured data for this study, a total of 15 152 patients with moderate and severe TBI met the inclusion criteria (Figure 1). Of these, 3576 (24%) experienced any type of infection based on relevant ICD‐10‐AM codes (Table 2). Eighty percent of codes with an ICD‐10‐AM code for infections were classified with a C‐prefix as a complicating condition acquired during hospitalization (79.6%). Most of the remaining cases were diagnosed with P‐prefix as primary condition presented at the time of admission (19.9%), and rarely with A‐prefix as an associated condition at the time of admission (0.2%) or U‐prefix as an unknown condition onset flag (0.3%). The proportion of infections classified as a complication was particularly high for meningitis, wound infections, pneumonia, intestinal infections, and sepsis (>85%), and was relatively lower for respiratory infections (63%), and urinary tract infections (72%). The details of types of infections reported are presented in Table 2.

**TABLE 2:** Summary of infection diagnosis.

Conditions	Diagnosis prefix, *n* (%)				Total, *n* (%)
	C	P	A	U	
All pneumonia	1344 (87)	192 (12)	3 (0.2)	2 (0.1)	1541 (29)
Urinary tract infections	662 (72)	252 (27)	3 (0.3)	7 (0.8)	924 (17)
All respiratory infections	548 (63)	320 (37)	2 (0.2)	3 (0.3)	873 (16)
Upper respiratory	14 (61)	7 (30)	2 (8.7)	0 (0)	23 (0.4)
Lower respiratory	534 (63)	313 (37)	0 (0)	3 (0.4)	850 (16)
Sepsis	527 (85)	90 (15)	1 (0.2)	1 (0.2)	619 (11)
Fever	487 (87)	70 (13)	2 (0.4)	1 (0.2)	560 (10)
Intestinal infections	277 (87)	41 (13)	0 (0)	0 (0)	318 (5.9)
Wound infections	98 (90)	11 (10)	0 (0)	0 (0)	109 (2.0)
Non‐specified bacterial infections	35 (78)	10 (22)	0 (0)	0 (0)	45 (0.8)
Meningitis	20 (91)	2 (9.1)	0 (0)	0 (0)	22 (0.4)
Post‐traumatic infections	3 (17)	15 (83)	0 (0)	0 (0)	18 (0.3)
Total	4001 (80)	1003 (19)	11 (0.2)	14 (0.3)	5029 (100)

Abbreviations: A, associate condition at the time of admission; C, complicating condition occurring after admission; P, primary condition at the time of admission; U, unknown condition onset flag.

Among the 3576 patients who experienced infection, 74.6% (*n* = 2666) had a single infection type, 19.6% (*n* = 702) had two infection types, and 5.8% (*n* = 208) had three or more types of infection. The most common combination was pneumonia and sepsis (*n* = 133; 14.6%), followed by pneumonia and urinary tract infections (*n* = 89; 9.8%), and pneumonia and respiratory infections (*n* = 71; 7.8%).

### 3.2 Incidence of PTE at 24 months follow‐up

After the PTE question was routinely asked at all follow‐ups from 12 March 2018, 1361 (73%) of the 1871 patients survived the first 24 months post‐injury were followed up at 24 months (Figure 1). Among them, 152 (11%) had developed PTE by 24 months post‐injury. Of these 152 patients, 45 (29.5%) were first recorded to have developed PTE at the 6‐month follow‐up time point. 19 patients (12.5%) reported PTE developing between 6–12 months and 34 (22.4%) between 12–24 months. A further 23 patients (15.1%) developed PTE any time within 12 months (as they missed the 6‐month follow‐up), and 21 (20.4%) developed PTE any time within 24 months (as they missed both the 6‐ and 12‐month follow‐ups).

Univariable analysis showed the proportion of PTE was higher among patients who had any type of complicating infection acquired during admission (20%) compared to patients that did not sustain an infection (9.2%; RR = 2.13; 95% CI: 1.56–2.90; *p* < 0.001) (Table 3).

**TABLE 3:** Univariable analysis of proportion of patients developed post‐traumatic epilepsy among the underlying complicating conditions.

Conditions	PTE among patients with the condition		PTE among patients without the condition		RR (95% CI)	*p*‐Value
	*n*	%	*n*	%		
Any infections	51/261	20	101/1100	9.2	2.13 (1.56–2.90)	<0.001
All pneumonia	25/120	21	127/1241	10	2.04 (1.38–2.99)	<0.001
Urinary tract infections	13/70	19	139/1291	11	1.72 (1.03–2.89)	0.038
All respiratory infections	11/48	23	141/1313	11	2.13 (1.24–3.67)	0.006
Fever	7/41	17	145/1320	11	1.55 (0.78–3.11)	0.21
Sepsis	7/40	18	145/1321	11	1.59 (0.80–3.18)	0.19
Intestinal infections	6/36	17	146/1325	11	1.51 (0.72–3.19)	0.28
Other infections^a^	0/6	0	152/1355	11	N/A	0.38

^a^ Other infections include meningitis, post‐traumatic, wound, and other mycobacteria infections that each had 5 or less patients. The *p*‐value was from chi‐square test.

Abbreviations: CI, confidence interval; N/A, not applicable; PTE, post‐traumatic epilepsy; RR, risk ratio.

Delving into infection subtypes, a higher proportion of patients with pneumonia had developed PTE by 24 months post‐injury (20%) compared to patients who did not sustain pneumonia (10%; RR = 2.04; 95% CI: 1.38–2.99; *p* < 0.001). This was also the case for respiratory infections (23% vs. 11%; RR = 2.13; 95% CI: 1.24–3.67; *p* = 0.006) and urinary tract infections (19% vs. 11%; RR = 1.72; 95% CI: 1.03–2.89; *p* = 0.038). Other infections or conditions were not associated with an increased risk of PTE (Table 3), although low sample numbers in these sub‐categories likely rendered the analyses underpowered.

The “infection burden,” categorized as 1 (*n* = 179) vs. 2 (*n* = 57) vs. ≥3 (*n* = 25) different infections, demonstrated no significant effects on the risk of PTE: 2 different infections (*n* = 13/57) vs. 1 (*n* = 36/179): RR = 1.13, 95% CI: 0.65–1.98, *p* = 0.66; ≥3 (*n* = 2/25) vs. 1 (*n* = 36/179): RR = 0.40, 95% CI: 0.10–1.55, *p* = 0.18; ≥3 (*n* = 2/25) vs. 2 (*n* = 13/57): RR = 0.35, 95% CI: 0.09–1.44, *p* = 0.15.

With adjustment for selected covariates (Table 4) in multivariable analysis, patients who experienced any type of complicating infection had a higher risk of developing PTE compared to patients who did not sustain an infection (adjusted RR = 1.59; 95% CI: 1.11–2.28; *p* = 0.011). Further multivariable analyses in infection subtypes with a *p* < 0.25 on univariable analysis did not find any infection subtypes associated with PTE (Table 5).

**TABLE 4:** Factors screened for their associations with infection and development of PTE using the purposeful variable selection method.

Factor	Univariable *p*‐value for association with infection	Univariable *p*‐value for association with development of PTE	Included in the initial model	Selected in the final model
Early posttraumatic seizures	<0.001	<0.001	Y	Y
Age	0.007	<0.001	Y	Y
ARIA category	0.13	0.092	Y	Y
Cause of injury – Low‐fall	0.22	<0.001	Y	Y
History of mental health conditions	<0.001	<0.001	Y	Y
Glasgow Coma Scale category	<0.001	<0.001	Y	Y
Pre‐existing Charlson Comorbidity Index category	<0.001	<0.001	Y	N
Cause of injury – Motorcycle	0.10	0.011	Y	N
History of alcohol misuse	0.024	0.053	Y	N
Subdural hematoma	0.016	0.17	Y	N
Subarachnoid hemorrhage	0.047	<0.001	Y	N
Contusion	0.12	0.001	Y	N
AIS head severity category	<0.001	<0.001	Y	N
Any neurosurgery	<0.001	<0.001	Y	N
Sex	0.39	0.16	N	N
IRSAD quintile	0.24	0.78	N	N
Cause of injury – MVA	0.76	<0.001	N	N
Cause of injury – Bicycle	0.77	0.044	N	N
Cause of injury – Pedestrian	0.59	<0.001	N	N
Cause of injury – High‐fall	0.40	0.21	N	N
Cause of injury – Others	0.54	<0.001	N	N
History of drug misuse	0.83	0.20	N	N
Trauma type	0.38	<0.001	N	N
Intracerebral hemorrhage	0.019	0.28	N	N
Intraventricular hemorrhage	0.83	<0.001	N	N
Epidural hematoma	0.25	0.98	N	N
Diffuse axonal injury	0.32	<0.001	N	N
Base of skull fracture	0.64	0.003	N	N
Vault fractures	0.59	0.89	N	N
Injury Severity Score category	0.69	0.027	N	N

Abbreviations: AIS, abbreviated injury scale; ARIA, accessibility and remoteness index of Australia; IRSAD, index of relative socio‐economic advantage and disadvantage.

**TABLE 5:** Multivariate analyses of associations between hospital‐acquired infection and post‐traumatic epilepsy.

	Adjusted RR (95% CI)	*p*‐Value
Any infection	1.59 (1.11–2.28)	0.011
*Infection subtypes*		
Fever	1.01 (0.49–2.11)	0.97
All pneumonia	1.41 (0.87–2.27)	0.16
Urinary tract infections	1.26 (0.71–2.22)	0.43
All respiratory infections	1.59 (0.93–2.73)	0.089
Sepsis	1.10 (0.48–2.50)	0.83

*Note*: All analyses were adjusted for early posttraumatic seizures, age, cause of injury – low‐fall, history of mental health conditions, Glasgow Coma Scale category, and ARIA category.

Abbreviations: ARIA, accessibility and remoteness index of Australia; CI, confidence interval; PTE, post‐traumatic epilepsy; RR, risk ratio.

Patients who had any type of complicating infection acquired during initial admission for TBI were also associated with poor GOSE outcomes at subsequent follow‐ups (adjusted odds ratio [OR] = 0.20; 95% CI: 0.11–0.35, *p* < 0.001), after adjusted for follow‐up time point, early posttraumatic seizures, age, sex, history of mental health conditions, and Glasgow Coma Scale category.

## 4 DISCUSSION

This study aimed to determine whether infections sustained during acute hospitalization following severe TBI were associated with the risk of PTE up to 2 years post‐injury. Secondarily, we sought to evaluate whether infections were associated with outcomes. We found through univariate analysis that the incidence of PTE was higher in patients who had sustained a hospital‐acquired infection (20%) compared to those that did not sustain an infection (9.2%), particularly for pneumonia, respiratory tract, and urinary tract infections. This association was a bit weaker yet remained significant by multivariate analysis (adjusted RR = 1.59; 95% CI: 1.11–2.28). Several past studies have indicated that injury severity measures such as ISS and head AIS are risk factors for the occurrence of ventilator‐associated pneumonia in patients with severe TBI,^43^, ^44^ suggesting that high injury severity has a considerable influence over one's susceptibility to infection.

Overall, the statistical significance of our findings indicates a solid association between infection and PTE that can be detected with the current sample size. It is in line with several older studies that reported a link between wound sepsis or CNS infection and seizures/epilepsy after penetrating head injuries in military personnel.^26^, ^27^ Ascroft (1941) examined the reports of World War I veterans who sustained gunshot wounds to the head. Of 317 cases, 34% reported one or more seizure “fits,” and these were twice as frequent when wound healing was delayed more than 60 days (indicating prolonged wound sepsis),^26^ compared to patients with wounds that healed in less than 60 days, or less than 15 days (the lowest incidence). This finding was independent of dural penetration, which in itself was associated with an increased risk of seizures.^26^ More recently, a retrospective study of 489 veterans with penetrating head injuries from the Iran‐Iraq War reported that 32% of the population developed epilepsy across a median follow‐up time of 39 months, and central nervous system (CNS) infections (intracranial sepsis) were independently associated with the risk of epilepsy by univariate analysis.^27^ Finally, a recent study from the University of Pittsburgh also found that intracranial infections were independently associated with the onset of the first late post‐traumatic seizure.^45^ Together, these findings suggest that intracranial or extracranial infections may be previously unrecognized predictors of PTE alongside established risk factors such as penetrating injury, depressed skull fracture, cerebral contusion, and early post‐traumatic seizures.^6^, ^7^, ^8^, ^9^, ^10^, ^45^, ^46^, ^47^, ^48^

It is well established that infection risk is high in the intensive care unit, particularly for patients with TBI who often present with multiple comorbidities.^13^ Indwelling catheters and arterial lines, neurosurgical procedures, ventilator use, and injury‐induced immunosuppression, all likely increase susceptibility to infection in this population.^34^, ^49^, ^50^ We found that 24% of patients with a severe TBI over a 15‐year reporting period also sustained an infection, with the vast majority of these (80%) recorded as being acquired during hospitalization. Consistent with previous literature,^13^, ^34^, ^35^ the most common complicating condition (i.e., reported as onset after hospital admission) was pneumonia, followed by urinary tract infections and other respiratory tract infections. Ventilator‐acquired pneumonia in TBI patients is an established risk factor for worsened outcomes, including increased morbidity, longer hospital stay, and increased need for rehabilitation compared to matched TBI patients without infections.^20^, ^51^, ^52^, ^53^ Our new findings provide some evidence to suggest that these infections may also drive secondary neuropathological mechanisms, such as inflammation, to promote epileptogenesis after TBI. Independent of TBI, it is increasingly understood that immune challenges such as bacterial and viral infections can promote neuronal network hyperexcitability and lead to seizures or epilepsy.^54^ For example, infections often trigger the release of pro‐inflammatory cytokines such as interleukin‐1 beta (IL‐1β). Preclinical evidence has shown that signaling via IL‐1β can enhance long‐term excitability following immune activation,^55^ including in the context of experimental TBI^56^; while biomarker studies have reported that IL‐1β is associated with the development of PTE.^11^ Our current findings support future studies to delineate the cellular and molecular mechanisms underlying this interaction in the context of infections, immune responses, and epilepsy following brain injuries.

There were some limitations to note regarding our approach. Firstly, we relied on recorded ICD codes to assess infection exposure, rather than positive microbiology cultures (not available), which may contribute to underreporting of infections or reporting bias. Secondly, epilepsy diagnosis at 2 years follow‐up was based on response to a self‐reporting questionnaire, as the only available information, and to our knowledge, the sensitivity and specificity of this questionnaire have not been established. Further, evidence that a single reported post‐TBI seizure developed into recurrent seizure (i.e., PTE) in our cohort was not available. A clinical diagnosis according to the most widely accepted classifications of epilepsy according to the International League Against Epilepsy^57^ would have been preferred if available in future studies. Further, the sample size was unavoidably restricted to a subset of available patients in the Registry who had routinely been asked this question at follow‐up since 2018, rather than using the entire dataset, thus limiting the statistical power.

We also did not have detailed information on pharmacological drug use in this population which may have influenced our findings, as it has been reported that as many as 90% of TBI patients with hospital‐acquired pneumonia receive antibiotic treatments,^58^ and prophylactic anti‐seizure medications may also be routinely administered.^59^ The study design did not allow us to determine whether an infection might alter the time course of PTE development – for example, it is feasible that an infection could accelerate the process of epileptogenesis after injury, resulting in a shorter latency to the onset of the first late post‐traumatic seizure.^60^ Finally, infections are a leading cause of potentially avoidable re‐admission episodes to acute care for TBI patients,^61^ and it remains unclear how or whether such post‐injury infection challenges during the chronic post‐injury period might influence the development of PTE. These unanswered questions should be addressed in future studies.

## 5 CONCLUSION

Our findings indicate that hospital‐acquired infections are associated with the development of PTE after severe TBI. This raises the question of whether infections represent a modifiable risk factor and therapeutic target to reduce or prevent epilepsy in this population. For example, systemic infections can be minimized through the implementation of non‐pharmacological infection control strategies,^62^ while acute post‐injury prophylactic broad‐spectrum antibiotics to treat infections have been associated with improved survival.^63^ Early screening for pneumonia in intubated trauma patients via assessment of bronchoalveolar lavage fluid has also been shown to reduce the incidence of ventilator‐associated pneumonia.^64^ Alongside previous reports of a high incidence of infections detected in patients with moderate and severe TBI, our findings suggest that minimizing the incidence of hospital‐acquired infections in patients after severe TBI as a means to potentially prevent poor outcomes including worse GOSE and the development of PTE in a subset of vulnerable individuals. Future studies in which a clinical diagnosis of epilepsy can be examined following a prior hospital‐acquired infection are warranted to confirm this relationship.

## AUTHOR CONTRIBUTIONS

Project conceptualization: Bridgette D. Semple, Terence J. O'Brien, Joshua Laing, Jian Li. Data access and analysis: Zhibin Chen, Joshua Laing, Belinda J. Gabbe. Manuscript draft: Zhibin Chen, Bridgette D. Semple, Belinda J. Gabbe. Manuscript edits: Zhibin Chen, Joshua Laing, Jian Li, Terence J. O'Brien, Belinda J. Gabbe, Bridgette D. Semple.

## CONFLICT OF INTEREST STATEMENT

None of the authors has any conflict of interest to disclose. The funders had no role in the design of the study; in the collection, analyses, or interpretation of data; in the writing of the manuscript; or in the decision to publish the results. We confirm that we have read the Journal's position on issues involved in ethical publication and affirm that this report is consistent with those guidelines.

## ETHICS STATEMENT

All authors have read the Journal's position on issues involved in ethical publication and we affirm that this report is consistent with those guidelines.

## Data Availability

The authors agree to provide the full content of the manuscript on request by contacting the corresponding author. The VSTR is governed by the VSTORM group, and access to the VSTR dataset requires approval from VSTORM. Data requests can be made via the following link: https://www.monash.edu/medicine/sphpm/vstorm/data‐requests. Sharing a de‐identified data set is not possible due to restrictions imposed by the Victorian Department of Health Ethics Committee, which have provided ethical approval for the collection and subsequent use of VSTR data.

## References

[1] Fisher RS , Acevedo C , Arzimanoglou A , Bogacz A , Cross JH , Elger CE , et al. ILAE official report: a practical clinical definition of epilepsy. Epilepsia. 2014;55(4):475–482. 24730690 10.1111/epi.12550

[2] Frey LC . Epidemiology of posttraumatic epilepsy: a critical review. Epilepsia. 2003;44(Suppl 10):11–17. 10.1046/j.1528-1157.44.s10.4.x 14511389

[3] Christensen J , Pedersen MG , Pedersen CB , Sidenius P , Olsen J , Vestergaard M . Long‐term risk of epilepsy after traumatic brain injury in children and young adults: a population‐based cohort study. Lancet. 2009;373(9669):1105–1110. 19233461 10.1016/S0140-6736(09)60214-2

[4] Englander J , Bushnik T , Duong TT , Cifu DX , Zafonte R , Wright J , et al. Analyzing risk factors for late posttraumatic seizures: a prospective, multicenter investigation. Arch Phys Med Rehabil. 2003;84(3):365–373. 12638104 10.1053/apmr.2003.50022

[5] Mazzini L , Cossa FM , Angelino E , Campini R , Pastore I , Monaco F . Posttraumatic epilepsy: neuroradiologic and neuropsychological assessment of long‐term outcome. Epilepsia. 2003;44(4):569–574. 12681007 10.1046/j.1528-1157.2003.34902.x

[6] Burke J , Gugger J , Ding K , Kim JA , Foreman B , Yue JK , et al. Association of posttraumatic epilepsy with 1‐year outcomes after traumatic brain injury. JAMA Netw Open. 2021;4(12):e2140191. 34964854 10.1001/jamanetworkopen.2021.40191 PMC8717106

[7] Khalili H , Kashkooli NR , Niakan A , Asadi‐Pooya AA . Risk factors for post‐traumatic epilepsy. Seizure. 2021;89:81–84. 34023655 10.1016/j.seizure.2021.05.004

[8] Karlander M , Ljungqvist J , Zelano J . Post‐traumatic epilepsy in adults: a nationwide register‐based study. J Neurol Neurosurg Psychiatry. 2021;92(6):617–621. 33687971 10.1136/jnnp-2020-325382 PMC8142432

[9] Yu T , Liu X , Sun L , Wu J , Wang Q . Clinical characteristics of post‐traumatic epilepsy and the factors affecting the latency of PTE. BMC Neurol. 2021;21(1):301. 34348691 10.1186/s12883-021-02273-x PMC8340486

[10] Laing J , Gabbe B , Chen Z , Perucca P , Kwan P , O'Brien TJ . Risk factors and prognosis of early posttraumatic seizures in moderate to severe traumatic brain injury. JAMA Neurol. 2022;79:334–341. 35188950 10.1001/jamaneurol.2021.5420 PMC8861899

[11] Diamond ML , Ritter AC , Failla MD , Boles JA , Conley YP , Kochanek PM , et al. IL‐1beta associations with posttraumatic epilepsy development: a genetics and biomarker cohort study. Epilepsia. 2015;56(7):991–1001. 26149793 10.1111/epi.13100

[12] Diamond ML , Ritter AC , Jackson EK , Conley YP , Kochanek PM , Boison D , et al. Genetic variation in the adenosine regulatory cycle is associated with posttraumatic epilepsy development. Epilepsia. 2015;56(8):1198–1206. 26040919 10.1111/epi.13044 PMC4523397

[13] Kourbeti IS , Vakis AF , Papadakis JA , Karabetsos DA , Bertsias G , Filippou M , et al. Infections in traumatic brain injury patients. Clin Microbiol Infect. 2012;18(4):359–364. 21851488 10.1111/j.1469-0691.2011.03625.x

[14] Dziedzic T , Slowik A , Szczudlik A . Nosocomial infections and immunity: lesson from brain‐injured patients. Crit Care. 2004;8(4):266–270. 15312209 10.1186/cc2828 PMC522830

[15] Fàbregas N , Torres A . Pulmonary infection in the brain injured patient. Minerva Anestesiol. 2002;68(4):285–290. 12024101

[16] Zhang X , Zhou H , Shen H , Wang M . Pulmonary infection in traumatic brain injury patients undergoing tracheostomy: predicators and nursing care. BMC Pulm Med. 2022;22(1):130. 35392885 10.1186/s12890-022-01928-w PMC8988413

[17] Shuman EK , Chenoweth CE . Urinary catheter‐associated infections. Infect Dis Clin North Am. 2018;32(4):885–897. 30241712 10.1016/j.idc.2018.07.002

[18] Corral L , Javierre CF , Ventura JL , Marcos P , Herrero JI , Mañez R . Impact of non‐neurological complications in severe traumatic brain injury outcome. Crit Care. 2012;16(2):R44. 22410278 10.1186/cc11243 PMC3681369

[19] Selassie AW , Fakhry SM , Ford DW . Population‐based study of the risk of in‐hospital death after traumatic brain injury: the role of sepsis. J Trauma. 2011;71(5):1226–1234. 22071924 10.1097/TA.0b013e318226ecfc

[20] Kesinger MR , Kumar RG , Wagner AK , Puyana JC , Peitzman AP , Billiar TR , et al. Hospital‐acquired pneumonia is an independent predictor of poor global outcome in severe traumatic brain injury up to 5 years after discharge. J Trauma Acute Care Surg. 2015;78(2):396–402. 25757128 10.1097/TA.0000000000000526 PMC5070940

[21] Esnault P , Nguyen C , Bordes J , D'Aranda E , Montcriol A , Contargyris C , et al. Early‐onset ventilator‐associated pneumonia in patients with severe traumatic brain injury: incidence, risk factors, and consequences in cerebral oxygenation and outcome. Neurocrit Care. 2017;27(2):187–198. 28432539 10.1007/s12028-017-0397-4

[22] Ahlers FS , Benros ME , Dreier JW , Christensen J . Infections and risk of epilepsy in children and young adults: a nationwide study. Epilepsia. 2019;60(2):275–283. 30577081 10.1111/epi.14626

[23] Vezzani A , Fujinami RS , White HS , Preux PM , Blümcke I , Sander JW , et al. Infections, inflammation and epilepsy. Acta Neuropathol. 2016;131(2):211–234. 26423537 10.1007/s00401-015-1481-5 PMC4867498

[24] Webster KM , Sun M , Crack P , O'Brien TJ , Shultz SR , Semple BD . Inflammation in epileptogenesis after traumatic brain injury. J Neuroinflammation. 2017;14(1):10. 28086980 10.1186/s12974-016-0786-1 PMC5237206

[25] Dube C , Brewster AL , Richichi C , Zha Q , Baram TZ . Fever, febrile seizures and epilepsy. Trends Neurosci. 2007;30(10):490–496. 17897728 10.1016/j.tins.2007.07.006 PMC2766556

[26] Ascroft PB . Traumatic epilepsy after gunshot wounds of the head. Br Med J. 1941;1(4193):739–744. 20783661 10.1136/bmj.1.4193.739 PMC2161603

[27] Aarabi B , Taghipour M , Haghnegahdar A , Farokhi M , Mobley L . Prognostic factors in the occurrence of posttraumatic epilepsy after penetrating head injury suffered during military service. Neurosurg Focus. 2000;8(1):e1. 10.3171/foc.2000.8.1.155 16906697

[28] Gandasasmita N , Li J , Loane DJ , Semple BD . Experimental models of hospital‐acquired infections after traumatic brain injury: challenges and opportunities. J Neurotrauma. 2023;41:752–770. 37885226 10.1089/neu.2023.0453

[29] Gabbe BJ , Braaf S , Cameron PA , Berecki‐Gisolf J . Epidemiology and 6‐ and 12‐month outcomes of intimate partner violence and other violence‐related traumatic brain injury in major trauma: a population‐based trauma registry study. J Head Trauma Rehabil. 2022;37(1):E1–e9. 34985035 10.1097/HTR.0000000000000741

[30] Foreman BP , Caesar RR , Parks J , Madden C , Gentilello LM , Shafi S , et al. Usefulness of the abbreviated injury score and the injury severity score in comparison to the Glasgow Coma Scale in predicting outcome after traumatic brain injury. J Trauma. 2007;62(4):946–950. 17426553 10.1097/01.ta.0000229796.14717.3a

[31] Demetriades D , Kuncir E , Murray J , Velmahos GC , Rhee P , Chan L . Mortality prediction of head Abbreviated Injury Score and Glasgow Coma Scale: analysis of 7,764 head injuries. J Am Coll Surg. 2004;199(2):216–222. 15275876 10.1016/j.jamcollsurg.2004.02.030

[32] Youngblut JM , Caicedo C , Brooten D . Preschool children with head injury: comparing injury severity measures and clinical care. Pediatr Nurs. 2013;39(6):290–298. 24640315 PMC4120249

[33] von Elm E , Altman DG , Egger M , Pocock SJ , Gøtzsche PC , Vandenbroucke JP . The Strengthening the Reporting of Observational Studies in Epidemiology (STROBE) statement: guidelines for reporting observational studies. Lancet. 2007;370(9596):1453–1457. 18064739 10.1016/S0140-6736(07)61602-X

[34] Rivera‐Lara L , Ziai W , Nyquist P . Management of infections associated with neurocritical care. Handb Clin Neurol. 2017;140:365–378. 28187810 10.1016/B978-0-444-63600-3.00020-9

[35] Wirtz MR , Moekotte J , Balvers K , Admiraal MM , Pittet JF , Colombo J , et al. Autonomic nervous system activity and the risk of nosocomial infection in critically ill patients with brain injury. Intensive Care Med Exp. 2020;8(1):69. 33237337 10.1186/s40635-020-00359-3 PMC7688871

[36] Gabbe BJ , Magtengaard K , Hannaford AP , Cameron PA . Is the Charlson Comorbidity Index useful for predicting trauma outcomes? Acad Emerg Med. 2005;12(4):318–321. 15805322 10.1197/j.aem.2004.12.002

[37] Nguyen TQ , Simpson PM , Braaf SC , Cameron PA , Judson R , Gabbe BJ . Comparison of the performance of mental health, drug and alcohol comorbidities based on ICD‐10‐AM and medical records for predicting 12‐month outcomes in trauma patients. BMC Health Serv Res. 2018;18(1):408. 29871639 10.1186/s12913-018-3248-x PMC5989374

[38] Wilson JT , Pettigrew LE , Teasdale GM . Structured interviews for the Glasgow Outcome Scale and the extended Glasgow Outcome Scale: guidelines for their use. J Neurotrauma. 1998;15(8):573–585. 9726257 10.1089/neu.1998.15.573

[39] Weiss GH , Salazar AM , Vance SC , Grafman JH , Jabbari B . Predicting posttraumatic epilepsy in penetrating head injury. Arch Neurol. 1986;43(8):771–773. 3089201 10.1001/archneur.1986.00520080019013

[40] Annegers JF , Hauser WA , Coan SP , Rocca WA . A population‐based study of seizures after traumatic brain injuries. N Engl J Med. 1998;338(1):20–24. 9414327 10.1056/NEJM199801013380104

[41] Haltiner AM , Temkin NR , Dikmen SS . Risk of seizure recurrence after the first late posttraumatic seizure. Arch Phys Med Rehabil. 1997;78(8):835–840. 9344302 10.1016/s0003-9993(97)90196-9

[42] Hosmer DWJ , Lemeshow S , May S . Applied survival analysis: regression modeling of time‐to‐event data. 2nd ed. Hoboken, NJ: Wiley; 2008.

[43] Li Y , Liu C , Xiao W , Song T , Wang S . Incidence, risk factors, and outcomes of ventilator‐associated pneumonia in traumatic brain injury: a meta‐analysis. Neurocrit Care. 2020;32(1):272–285. 31300956 10.1007/s12028-019-00773-w PMC7223912

[44] Jovanovic B , Milan Z , Markovic‐Denic L , Djuric O , Radinovic K , Doklestic K , et al. Risk factors for ventilator‐associated pneumonia in patients with severe traumatic brain injury in a Serbian trauma centre. Int J Infect Dis. 2015;38:46–51. 26166697 10.1016/j.ijid.2015.07.005

[45] Pease M , Gonzalez‐Martinez J , Puccio A , Nwachuku E , Castellano JF , Okonkwo DO , et al. Risk factors and incidence of epilepsy after severe traumatic brain injury. Ann Neurol. 2022;92(4):663–669. 35713346 10.1002/ana.26443 PMC9489614

[46] Thijs RD , Surges R , O'Brien TJ , Sander JW . Epilepsy in adults. Lancet. 2019;393(10172):689–701. 30686584 10.1016/S0140-6736(18)32596-0

[47] Raymont V , Salazar AM , Lipsky R , Goldman D , Tasick G , Grafman J . Correlates of posttraumatic epilepsy 35 years following combat brain injury. Neurology. 2010;75(3):224–229. 20644150 10.1212/WNL.0b013e3181e8e6d0 PMC2906177

[48] Wang H , Xin T , Sun X , Wang S , Guo H , Holton‐Burke C , et al. Post‐traumatic seizures ‐ a prospective, multicenter, large case study after head injury in China. Epilepsy Res. 2013;107:272–278. 24239245 10.1016/j.eplepsyres.2013.10.006

[49] Hu PJ , Pittet JF , Kerby JD , Bosarge PL , Wagener BM . Acute brain trauma, lung injury, and pneumonia: more than just altered mental status and decreased airway protection. Am J Physiol Lung Cell Mol Physiol. 2017;313(1):L1–l15. 28408366 10.1152/ajplung.00485.2016

[50] Sharma R , Shultz SR , Robinson MJ , Belli A , Hibbs ML , O'Brien TJ , et al. Infections after a traumatic brain injury: the complex interplay between the immune and neurological systems. Brain Behav Immun. 2019;79:63–74. 31029794 10.1016/j.bbi.2019.04.034

[51] Hamele M , Stockmann C , Cirulis M , Riva‐Cambrin J , Metzger R , Bennett TD , et al. Ventilator‐associated pneumonia in pediatric traumatic brain injury. J Neurotrauma. 2016;33(9):832–839. 26203702 10.1089/neu.2015.4004 PMC4939445

[52] Hofman M , Andruszkow H , Kobbe P , Poeze M , Hildebrand F . Incidence of post‐traumatic pneumonia in poly‐traumatized patients: identifying the role of traumatic brain injury and chest trauma. Eur J Trauma Emerg Surg. 2020;46(1):11–19. 31270555 10.1007/s00068-019-01179-1 PMC7223163

[53] Kumar RG , Kesinger MR , Juengst SB , Brooks MM , Fabio A , Dams‐O'Connor K , et al. Effects of hospital‐acquired pneumonia on long‐term recovery and hospital resource utilization following moderate to severe traumatic brain injury. J Trauma Acute Care Surg. 2020;88(4):491–500. 31804412 10.1097/TA.0000000000002562 PMC7802881

[54] Vezzani A , Balosso S , Ravizza T . Neuroinflammatory pathways as treatment targets and biomarkers in epilepsy. Nat Rev Neurol. 2019;15(8):459–472. 31263255 10.1038/s41582-019-0217-x

[55] Auvin S , Shin D , Mazarati A , Sankar R . Inflammation induced by LPS enhances epileptogenesis in immature rat and may be partially reversed by IL1RA. Epilepsia. 2010;51(Suppl 3):34–38. 20618397 10.1111/j.1528-1167.2010.02606.x PMC2910518

[56] Semple BD , O'Brien TJ , Gimlin K , Wright DK , Kim SE , Casillas‐Espinosa PM , et al. Interleukin‐1 receptor in seizure susceptibility after traumatic injury to the pediatric brain. J Neurosci. 2017;37(33):7864–7877. 28724747 10.1523/JNEUROSCI.0982-17.2017 PMC5559762

[57] Scheffer IE , Berkovic S , Capovilla G , Connolly MB , French J , Guilhoto L , et al. ILAE classification of the epilepsies: position paper of the ILAE Commission for Classification and Terminology. Epilepsia. 2017;58(4):512–521. 28276062 10.1111/epi.13709 PMC5386840

[58] Beghi G , De Tanti A , Serafini P , Bertolino C , Celentano A , Taormina G . Monitoring of hospital acquired pneumonia in patients with severe brain injury on first access to intensive neurological rehabilitation: first year of observation. Monaldi Arch Chest Dis. 2018;88(1):888. 29741076 10.4081/monaldi.2018.888

[59] Torbic H , Forni AA , Anger KE , Degrado JR , Greenwood BC . Use of antiepileptics for seizure prophylaxis after traumatic brain injury. Am J Health‐Syst Pharm. 2013;70(9):759–766. 23592358 10.2146/ajhp120203

[60] Semple BD , Dill LK , O'Brien TJ . Immune challenges and seizures: how do early life insults influence epileptogenesis? Front Pharmacol. 2020;11:2. 32116690 10.3389/fphar.2020.00002 PMC7010861

[61] McKechnie D , Fisher MJ , Pryor J , Mckechnie R . An examination of the nature and characteristics of patients readmitted to acute care from inpatient brain injury rehabilitation. J Adv Nurs. 2020;76(10):2586–2596. 32748979 10.1111/jan.14475

[62] Curtis LT . Prevention of hospital‐acquired infections: review of non‐pharmacological interventions. J Hosp Infect. 2008;69(3):204–219. 18513830 10.1016/j.jhin.2008.03.018 PMC7172535

[63] Dhillon NK , Adjamian N , Fierro NM , Conde G , Barmparas G , Ley EJ . Early antibiotic administration is independently associated with improved survival in traumatic brain injury. J Surg Res. 2022;270:495–502. 34808469 10.1016/j.jss.2021.10.015

[64] Harrell KN , Lee WB , Rooks HJ , Briscoe WE , Capote W , Dart BW , et al. Early pneumonia diagnosis decreases ventilator‐associated pneumonia rates in trauma population. J Trauma Acute Care Surg. 2022;94:30–35. 36245076 10.1097/TA.0000000000003808

